# Clinical Insights and Therapeutic Strategies for the Treatment of Interstitial Lung Disease in Patients with Anti-Neutrophil Cytoplasmic Antibody-Associated Vasculitis: Current Trends and Future Directions

**DOI:** 10.3390/jcm14134631

**Published:** 2025-06-30

**Authors:** Justyna Fijolek, Anna Sniady

**Affiliations:** 1 Third Department of Pneumonology and Oncology, National Tuberculosis and Lung Diseases Research Institute, 01-138 Warsaw, Poland; 2 Medical Academy of Applied Holistic Sciences, 01-234 Warsaw, Poland; anna.sniady22@gmail.com

**Keywords:** ANCA-associated vasculitis, interstitial lung disease, ANCA-positive ILD, pulmonary fibrosis, microscopic polyangiitis, immunosuppressive therapy, antifibrotic agents, personalized treatment

## Abstract

Anti-neutrophil cytoplasmic antibody (ANCA)-associated vasculitis (AAV) and interstitial lung disease (ILD) represent a complex interplay between autoimmune and fibrotic processes that poses significant diagnostic and therapeutic challenges. The distinction between isolated ANCA-ILD and AAV-ILD remains a subject of ongoing debate, with some researchers proposing that ANCA-ILD may be an early or restricted form of systemic vasculitis. Immunosuppressive therapy is the cornerstone of treatment for both diseases. However, there is increasing evidence that supports the potential role of antifibrotic agents in the management of progressive fibrosis. Management of these diseases requires a personalized approach that incorporates evaluation of biomarkers, imaging findings, and clinical risk factors to guide treatment decisions. Although current therapeutic strategies primarily target systemic inflammation, addressing the fibrotic components of these diseases is crucial for improving outcomes. Furthermore, emerging therapeutic options, such as B-cell depletion and antifibrotic therapies, offer promising outcomes. However, their roles in the treatment of AAV-ILD require further exploration. In this review, we discuss clinical insights and evolving therapeutic strategies for managing AAV and ANCA-positive ILD. In addition, we highlight the importance of early diagnosis and individualized treatment plans in improving the prognosis and quality of life of affected patients.

## 1. Introduction

Anti-neutrophil cytoplasmic antibody (ANCA)-associated vasculitides (AAVs) are a heterogeneous group of rare primary disorders characterized by necrotizing inflammation of small- and medium-sized blood vessels. From a clinicopathological perspective, AAVs can be categorized into four distinct entities: granulomatosis with polyangiitis (GPA), microscopic polyangiitis (MPA), eosinophilic granulomatosis with polyangiitis (EGPA), and renal-limited vasculitis [1]. Although not pathognomonic, a hallmark feature of AAVs is the presence of ANCA directed against neutrophil granule components, namely: proteinase 3 (PR3-ANCA), which is more frequently observed in GPA, and myeloperoxidase (MPO-ANCA), which is more commonly found in MPA, EGPA, and renal-limited vasculitis [2]. However, not all patients with AAV have detectable ANCA, particularly those with EGPA, in whom seropositivity is observed in only 30–40% of cases [3,4], and even less frequently among patients presenting with a predominantly tissue-based phenotype [5]. Since ANCA serology is correlated with distinct clinical phenotypes linked to genetic predisposition, current classification trends favor distinguishing AAVs based on ANCA specificity (MPO-AAV and PR3-AAV) rather than traditional clinical syndromes [6]. This approach does not apply to EGPA, which markedly differs from GPA and MPA in terms of pathophysiology and clinical features and has usually been examined as a distinct entity [7].

The manifestations of AAVs are diverse, with a predilection for the kidneys, respiratory system, peripheral nervous system, and skin [2]. Pulmonary involvement is common and presents as masses, cavities, and nodules; diffuse alveolar hemorrhage (DAH); large airway disease (subglottic stenosis and tracheobronchial inflammation); and interstitial lung disease (ILD). In contrast, EGPA is typically characterized by migrating eosinophilic infiltrates consistent with eosinophilic pneumonia [8]. The clinical presentation of pulmonary involvement in AAVs is heterogeneous, ranging from incidental findings on imaging studies to life-threatening complications requiring mechanical ventilation [2,9].

ILD is characterized by inflammation and/or fibrosis within the alveolar interstitium of the lungs and encompasses a broad spectrum of disorders [10,11]. The association between ILD and AAV was first observed in 1990 in three elderly patients with an initial diagnosis of idiopathic pulmonary fibrosis (IPF), who were later diagnosed with pulmonary-renal syndrome and MPA [12]. Since then, a steadily increasing number of studies have been conducted to examine this relationship between ILD and AAV. However, most of the studies were case series or retrospective analyses. Interestingly, cases of ILD with ANCA positivity, but without clear clinical features of AAV, have also been documented [13,14]. However, the differences between isolated ANCA-positive ILD (ANCA-ILD) and ILD secondary to AAV (AAV-ILD) remain unclear [15]. Moreover, some researchers have suggested that ANCA-ILD may be the only manifestation of MPA [16], analogous to GPA, in which a limited form is well recognized [17].

The presence of ILD in AAVs is associated with poorer prognosis [18]. Therefore, early diagnosis and prompt initiation of treatment are crucial for improving patient outcomes. Despite the increasing awareness of the association between ILD and AAVs, optimal management strategies for AAV-ILD remain unclear. In addition, treatment decisions are often based on clinical experience and data from the management of other forms of ILDs. In this review, we discuss the epidemiology, clinical manifestations, diagnosis, and treatment of ILD in AAVs, considering the current therapeutic strategies and the evolving landscape of future treatment perspectives. In addition, we discuss the clinical relevance of ANCA in ILD, including the potential for progression to systemic vasculitis, and whether isolated ANCA-positive ILD is a distinct phenotype within the AAV spectrum or a separate clinical entity. This review focuses on classical forms of ILD associated with AAV, primarily seen in MPA and GPA, acknowledging that ILD is extremely rare in EGPA, which represents a distinct clinical and pathophysiological entity and will not be addressed in this review.

## 2. Epidemiology

The prevalence of AAVs ranges from 300 to 421 cases per million individuals [19,20]. Although pulmonary involvement is common across all AAV subtypes, ILD is observed more frequently in patients with MPO-ANCA-positive MPA, with a documented prevalence of up to 51% in this subgroup, compared to up to 23% in patients with GPA [15,21,22,23,24,25,26,27,28,29] and only 6.3% in those with EGPA [30]. Notably, in a recently published Polish study of patients with GPA who were predominantly PR3-ANCA positive, no case of ILD was identified at baseline [31], which is consistent with the notion that ILD is more commonly associated with MPO-ANCA-positive AAV rather than PR3-ANCA-positivity, and may also reflect cohort-specific or geographic differences [31]. The incidence of ILD in patients with AAV is estimated to be 10–20 cases per million in the general population [32]. In addition, MPO-ANCA positivity is observed more frequently (ranging from 1.7% to 71%) in patients with ILD than PR3-ANCA positivity (0% to 29%) [14,23,27,33,34,35,36,37,38].

The temporal relationship between ANCA positivity, ILD, and the onset of systemic vasculitis is often complex. ANCA may be detected prior to ILD diagnosis (in 14–85% of cases), at the time of ILD diagnosis (36–67%), or may appear later during follow-up (8–21%) [14,24,28,35,39,40,41,42,43]. Conversely, seroconversion to ANCA positivity in patients initially diagnosed with idiopathic ILD has been reported in 2.63% to 14.8% of cases, with an estimated annual incidence of approximately 2% [12,18,33,35,44]. The interval between ILD diagnosis and the development of systemic vasculitis varies widely, from a few months to over a decade. However, most cases occur within the first 2 years [18,43,44].

Patients with AAV-ILD are typically diagnosed in their late 60s, with a slight male predominance noted in some studies [14,23,24,25,28,39,45]. In contrast, MPA without ILD is generally diagnosed approximately a decade earlier [14,24,25,28]. Although the results of several studies suggest a higher prevalence of AAV-ILD in Asian populations than in Western cohorts [24,25,28,37], significant differences in the overall prevalence of AAV between European and Japanese patients have not been demonstrated in prospective studies [24,25,28,46]. Notably, MPO-ANCA is more commonly detected in Asian patients, whereas PR3-ANCA is predominantly detected in European populations [22,24,25,28,37,46,47]. Consequently, AAV-ILD, which is strongly associated with MPO-ANCA, is reported more frequently in Asian cohorts [27,32,37].

## 3. Pathogenesis

**Figure 1:**
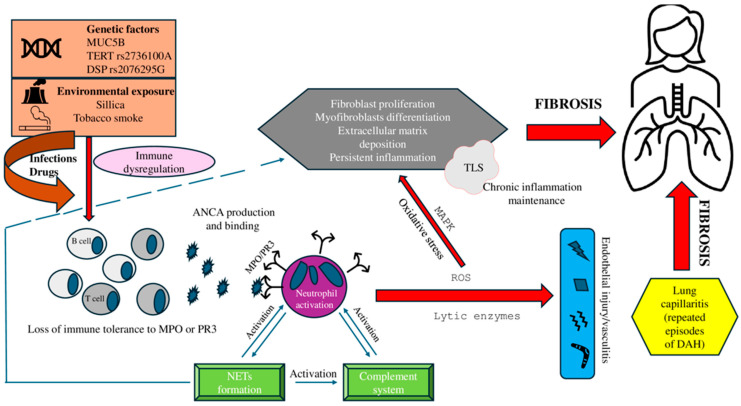
Pathogenesis of AAV-ILD. The pathogenesis of AAV-ILD involves complex interactions between environmental triggers and genetic susceptibility. These factors prime neutrophils, exposing MPO or PR3 on their surface and leading to loss of immune tolerance and ANCA production. ANCA binding activates neutrophils, causing the release of ROS and fibroblast proliferation, promoting interstitial fibrosis. NET formation upon stimulation of ANCA amplifies inflammation, damages the endothelium, and contributes to pulmonary capillaritis and DAH. Recurrent DAH fosters fibrosis through persistent inflammation and abnormal repair. NETs also activate fibroblasts into myofibroblasts, sustaining inflammation and extracellular matrix deposition, while serving as autoantigen sources that perpetuate autoimmunity. This chronic response is reinforced by TLS in the lung, which supports local autoantibody production and immune activation, perpetuating disease progression.

The pathogenesis of ILD in AAV is multifactorial and remains unclear (Figure 1). The proposed mechanisms include MPO-ANCA-induced oxidative damage and fibroblast activation [48], neutrophil extracellular trap (NET)-mediated myofibroblast transition [49], and chronic inflammation sustained by tertiary lymphoid structures (TLS) [50]. Recurrent subclinical DAH may also contribute to fibrosis [15,42,51]. ILD precedes the onset of AAV in some cases, possibly promoting the formation of MPO-ANCA through chronic neutrophil activation [22].

Recent studies have highlighted genetic factors associated with the risk of ILD in AAV. A Japanese study demonstrated that mucin 5B (MUC5B) promoter polymorphism is associated with an increased risk of ILD, more severe lung involvement, and increased pulmonary fibrosis in patients with MPO-ANCA vasculitis [52]. Another study revealed elevated IPF risk alleles (TERT rs2736100A and DSP rs2076295G) in patients with MPA/MPO-AAV. However, no significant association between allele frequencies was observed in patients with MPO-AAV with or without ILD [53].

## 4. Diagnosis

**Figure 2:**
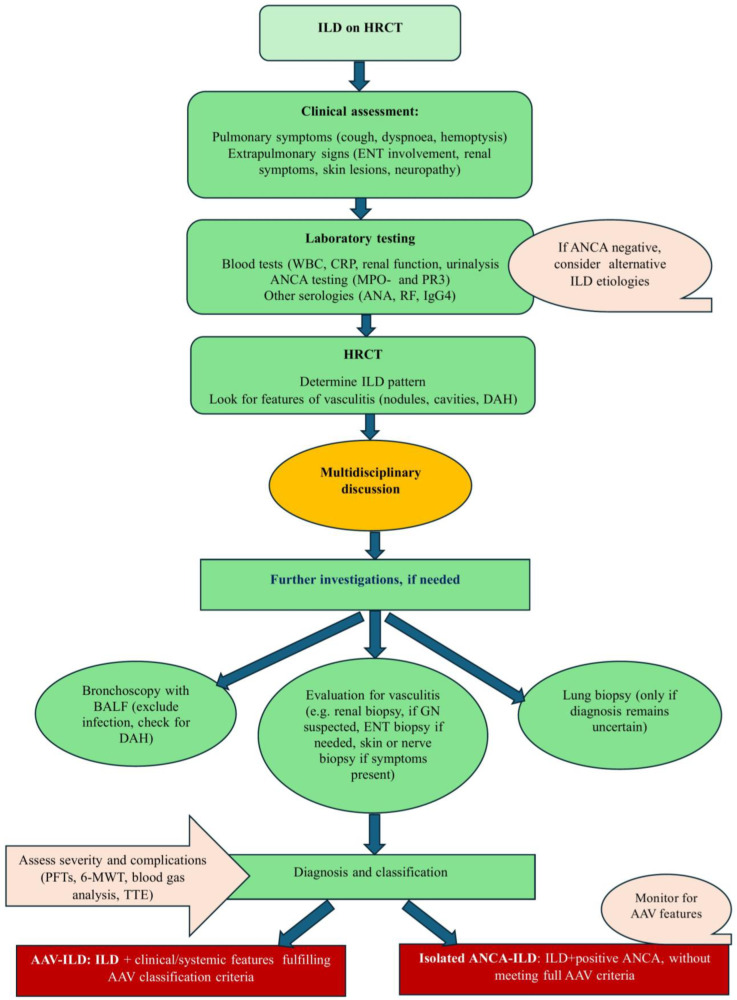
The diagram illustrates a stepwise diagnostic approach to the management of ANCA-ILD and AAV-ILD, beginning with the detection of interstitial lung abnormalities on HRCT. This is followed by laboratory testing, including ANCA serology, and assessment of HRCT patterns along with radiological features suggestive of pulmonary vasculitis (such as ground-glass opacities, nodules, or cavitation). A multidisciplinary discussion is recommended to guide decisions on further diagnostic procedures, such as bronchoscopy with BALF (to exclude infection or DAH) or lung biopsy when indicated. Final classification (AAV-ILD or isolated ANCA-ILD) is determined based on the presence or absence of systemic involvement, with ongoing monitoring for potential development of AAV. The algorithm also incorporates the evaluation of severity and ILD-related complications, which are crucial for accurate diagnosis and further management.

A multidimensional approach that integrates clinical, radiological, physiological, and, when necessary, histological findings is required for accurate diagnosis of ILD in patients with AAV and ANCA-positivity [11] (Figure 2). Notably, early diagnosis is crucial for timely initiation of treatment and improved outcomes.

### 4.1. Clinical Features

The clinical presentation of AAV-ILD is typically nonspecific, with dyspnea, exertional intolerance, and dry cough being the most common symptoms. In addition, inspiratory crackles suggestive of fibrosis are often detected during physical examinations. Systemic features such as hemoptysis, fever, or weight loss, reflecting systemic inflammation secondary to vasculitis, may occur [9,22,23,43,44]. However, they are less common in AAV-ILD than in AAV without ILD, which more frequently presents with cutaneous, ear, nose, throat (ENT), neurologic, cardiac, or gastrointestinal involvement [54]. Baseline disease activity in AAV-ILD tends to be lower than that in isolated AAV [23]. As with AAV-ILD, the clinical symptoms of ANCA-ILD are nonspecific and include dyspnea and dry cough. Although weight loss or fever has occasionally been reported, ANCA-ILD has no typical systemic or extrapulmonary features [55].

### 4.2. Serology and Laboratory Studies

ANCA is an important biomarker used in the diagnosis and classification of AAVs [56]. Although ANCA positivity is not strictly required for a diagnosis, testing is recommended for all patients with clinical features suggestive of AAV [57], as well those with ILD of unknown etiology [58], due to its potential to indicate future systemic disease [35]. Antigen-specific immunoassays targeting PR3 and MPO are preferred [57] because distinguishing between PR3-ANCA and MPO-ANCA has prognostic and therapeutic relevance, including indication of differences in the risk of relapse and survival [59,60]. A commonly used threshold for ANCA positivity is a titer ≥ four times the upper limit of normal, which has a reported sensitivity of 83.5% and specificity of 78.6% [61].

In addition to ANCA testing, routine laboratory workups may indicate signs of systemic inflammation or organ involvement, such as impaired renal function, anemia, and elevated levels of inflammatory markers. Notably, several emerging biomarkers are currently being studied for their potential role in the assessment and monitoring of patients with AVV [62,63,64,65]. Although overt systemic inflammation is often absent in patients with ANCA and ILD, they frequently test positive in screening tests for other autoantibodies, most commonly antinuclear antibodies and the rheumatoid factor (RF) [55].

### 4.3. Pulmonary Function Tests

Pulmonary function tests (PFTs) are essential in the evaluation of ILD. In a recent study, lung function abnormalities were present in up to 70% of patients at the time of diagnosis and were more frequently observed in patients with isolated ANCA-ILD than in those with AAV-ILD (76.9% vs. 40%) [44]. During follow-up (median: 36 months), functional decline was more common in the AAV-ILD group than in the ANCA-ILD group (50% vs. 23%). However, the transfer factor of the lung for carbon monoxide (TL,co) decreased similarly in both groups [44]. Reduced TL,co and restrictive defects are predominantly observed due to the characteristic fibrotic pattern of ILD. However, obstructive changes may also occur [14,25,27,28,35,42,66]. Notably, elevated TL,co may be an early sign of DAH [67].

### 4.4. Imaging

**Figure 3:**
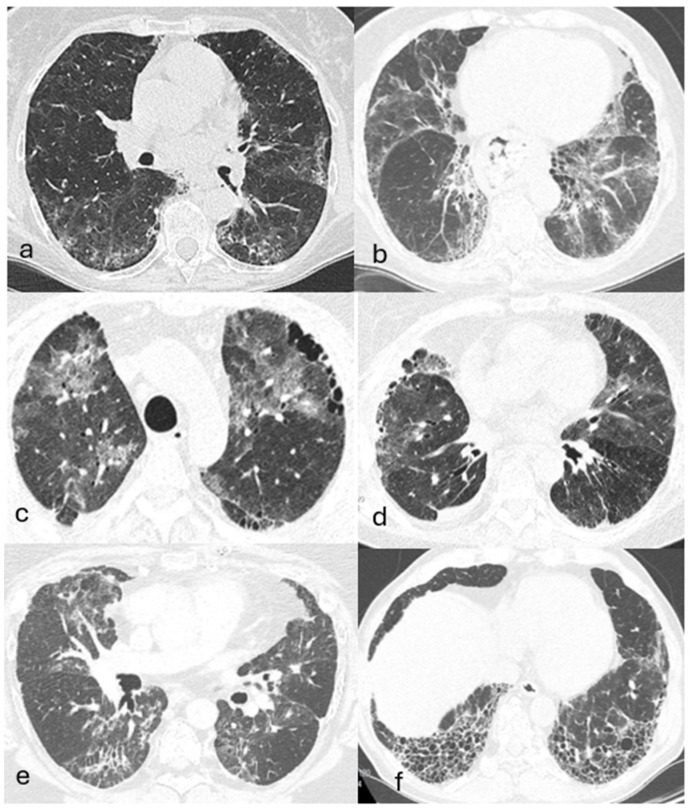
(**a**,**b**) HRCT scans show an NSIP pattern in the course of isolated ANCA-MPO-ILD. Bilateral ground–glass opacities, reticulation, and traction bronchiectasis are visible, with typical subpleural sparing and preserved volume in the lower lobes. Small subpleural honeycomb-like areas are also visible, which, although rare, can be observed in NSIP. (**c**,**d**) Bilateral interstitial lung changes in MPO-AAV with ground-glass and linear band-like opacities. Small subpleural honeycombing areas are also visible, along with volume loss in the right lower lobe and pleural thickening on the right side. (**e**) ILD in the course of PR3-AAV. Scan shows bilateral ground-glass opacities, band-like reticular abnormalities, and traction bronchiectasis, without evidence of honeycombing. (**f**) Bilateral honeycombing areas predominantly involving the lower lung fields, consistent with a UIP pattern.

Imaging, particularly high-resolution computed tomography (HRCT), is key to diagnosing AAV-associated and isolated ANCA-ILD. Typical findings include ground-glass (23–94%) and reticular opacities (41–77%), interlobular septal thickening (16–71%), consolidations (12–78%), honeycombing (23–83%), traction bronchiectasis (42–82.6%), and nodules (8.7–34%) [9,21,24,25,27,28,42,44,54,68,69,70]. In 4–40% of cases, HRCT reveals nonspecific interstitial abnormalities that do not meet the criteria for a defined ILD pattern [24,25,28,42]. The most predominantly observed pattern is usual interstitial pneumonia (UIP), which is detected in up to 74.5% of cases and is more often associated with MPO-ANCA than PR3-ANCA [70,71,72]. Non-specific interstitial pneumonia (NSIP) is the next most frequent pattern (7–31%), with cellular NSIP (9.5–26%) being more common than the fibrotic form (1–7%) [23,25,42,43,44,73] (Figure 3). Other patterns include organizing pneumonia (OP), desquamative interstitial pneumonia (DIP), NSIP/OP overlap, lymphoid interstitial pneumonia, and combined pulmonary fibrosis with emphysema [23,42,44,70,73].

Artificial intelligence-based tools improve the interpretation of AAV-ILD findings on HRCT by providing a consistent and quantitative assessment of key features [73,74]. In addition, radiomics may help distinguish AAV-ILD from other ILDs [74,75], whereas multimodal models that integrate CT and histology achieve high diagnostic accuracy (AUC: 0.92) and enhance pathologists’ confidence [76].

### 4.5. Bronchoscopy

Bronchoscopy with bronchoalveolar lavage fluid (BALF) analysis is not typically used in the diagnosis of ILD in AAV because the BALF findings are nonspecific. Neutrophilia is commonly observed, particularly in fibrosing subtypes, whereas lymphocytosis is less common [27]. Reports have indicated that patients with ANCA-ILD show higher neutrophil counts than those with ANCA-negative ILD [77]. In one study, hemosiderin-laden macrophages were identified in up to 70% of patients with AAV-related pulmonary fibrosis [42]. Despite its limited specificity, BALF remains essential for diagnosing or excluding DAH and ruling out infections or malignancies [11].

### 4.6. Biopsy and Histopathology

Histopathological evaluation of patients with AAV-ILD remains important, especially when HRCT findings are inconclusive. Although transbronchial biopsies are often non-diagnostic [78], surgical lung biopsy offers a high diagnostic yield but with substantial risk [79]. Transbronchial cryobiopsy is a valuable alternative that combines good diagnostic performance with a lower complication rate when performed in expert centers and guided by multidisciplinary discussions [80].

However, the pulmonary pathology of AAVs is heterogeneous and often challenging. GPA is characterized by necrotizing granulomatous inflammation and vasculitis [51], whereas MPA typically presents with pulmonary capillaritis without granulomas [50]. Histopathological patterns in AAV-ILD include acute injury (e.g., diffuse alveolar damage, acute fibrinous OP, and DAH) and chronic fibrosing changes (e.g., UIP, fibrotic NSIP, and fibrotic OP) [43]. UIP is the most common pattern, observed in more than half of patients with AAV-ILD [41,81], and often accompanied by inflammatory changes such as lymphoid follicles or cellular bronchiolitis, which are not typical of idiopathic UIP [77].

The available data on isolated ANCA-ILD are limited. In one study, eight of nine biopsies revealed a UIP pattern, often accompanied by NSIP features, small airway disease, and lymphoid follicles. Notably, no evidence of vasculitis was found in any case [82]. Similarly, in a study of 18 patients with MPO-ANCA-ILD, UIP was the most common pattern observed (56%) and often included bronchiolitis, lymphoid hyperplasia, DIP, and OP. NSIP was observed in 28% of the patients, and only one patient had granulomatous inflammation [71].

### 4.7. Disease Burden and Complications

In addition to pulmonary function testing, a thorough evaluation of ILD severity should include screening for pulmonary hypertension, preferably with transthoracic echocardiography (TTE), assessment of gas exchange to detect hypoxemia and potential respiratory insufficiency, and evaluation of functional capacity using the 6 min walk test (6-MWT). Identification of relevant comorbidities, including cardiovascular disease, gastroesophageal reflux disease (GERD), and obstructive sleep apnea, is essential because they may adversely impact disease trajectory and survival [10,11].

## 5. Phenotypes

Emerging data support the presence of two distinct AAV-ILD phenotypes. The first is characterized by ILD preceding the onset of AAV, typically associated with pANCA/MPO-ANCA seropositivity and a predominant UIP pattern on HRCT, often initially misclassified as IPF. The second phenotype is ILD diagnosed concurrently with or following AAV, more frequently presenting with an NSIP pattern and cANCA/PR3-ANCA or ANCA negativity. These findings underscore the need for longitudinal surveillance for AAV in patients with idiopathic ILD and for ILD in patients with AAV, particularly GPA [83].

## 6. Outcomes and Prognostic Factors

Although the outcomes of AAV have improved, the presence of ILD, particularly with a UIP pattern, is associated with a significantly elevated risk of mortality [24,84]. In one study, AAV-ILD conferred a 2.9-fold increased risk of death compared to AAV without ILD. In addition, UIP was associated with the highest risk of mortality among the ILD subtypes (RR, 4.36 vs. 2.90 in non-UIP ILD) [18].

In a study that included 80 patients with MPA-ILD (mostly with UIP), the 5-year survival rate was 58% versus 93% in those without ILD (*p* = 0.02). In addition, ILD independently increased the risk of respiratory-related death (HR, 7.2; *p* = 0.01) [85]. In another cohort of 62 patients with AAV-ILD (89% MPO-ANCA-positive), the 1-, 3-, and 5-year survival rates in the MPA-ILD group were lower than those in the non-ILD group, especially in patients with UIP, aged > 65 years, or with DAH at baseline [54]. A Chinese study of 204 patients with AAV-ILD confirmed the worst outcomes in patients with UIP, who had an approximately five-fold increased risk of mortality compared to those with NSIP [73]. Similarly, in a large retrospective cohort (n = 684), 13% of the patients had ILD (93% MPO-ANCA-positive), and mortality was higher in the ILD group (38% vs. 25%; *p* = 0.005), with a 39% increased risk of death after adjusting for age and sex, and 58% when all fibrotic patterns were included. Moreover, this association remained significant after adjustment for disease activity and renal involvement [23].

Several prognostic factors for AAV-ILD have been identified. In addition to the presence of a UIP pattern, poorer outcomes are associated with older age and DAH at baseline [54], as well as respiratory failure, weight loss, corticosteroid (CS) monotherapy [42], infections that require hospitalization [86], and elevated inflammatory markers [32]. In addition, reduced TL,co, with a proposed cutoff of 54.05%, is an adverse prognostic marker in AAV-ILD (HR, 0.970; *p* = 0.019) [86]. Similarly, a fibrosis score ≥ 2 in the lower lobes predicts poorer survival [85]. Moreover, increased neutrophil count in BALF is associated with an approximately 20% higher risk of mortality per 10% increment [73]. Finally, a recent meta-analysis that included 654 patients with AAV-ILD confirmed that older age, smoking, UIP pattern, disease exacerbation, and an MPA diagnosis are associated with an increased risk of mortality, whereas higher forced vital capacity (FVC) and immunosuppressive (IS) therapy are associated with improved survival. In contrast, male sex, nervous system or renal involvement, and a baseline five factor score ≥ 1 were not found to be significant predictors of mortality [87].

Interestingly, although most studies indicate a poorer prognosis in patients with AAV-ILD, particularly those with a UIP pattern, some data challenge this finding. Arulkumaran et al. [25] found no differences in survival between patients with MPA with and without ILD. However, the ILD subgroup in their study was small. Similarly, in a study of 206 patients with AAV, ILD was associated with a non-significant trend toward worse outcomes [88]. In contrast, a large Mayo Clinic study (1862 patients with AAV, 143 with ILD) indicated better survival in patients with MPO-ANCA with a UIP pattern than in those with other ILD patterns, suggesting differences in the mechanisms underlying the development of ILD in MPO-AAV versus PR3-AAV [72]. One study on the association between ILD and the risk of relapse in patients with AAV showed no significant difference between patients with AAV-ILD and those without ILD [54].

## 7. Isolated ANCA-Positive ILD: A Distinct AAV Phenotype or a Separate Entity? Clinical Significance of ANCA in ILD and the Risk of Developing AAV

A subset of patients with ILD, most commonly those with a pattern consistent with UIP, exhibits ANCA positivity in the absence of overt clinical vasculitis. This serological finding is most frequently associated with MPO-ANCA, which has been reported in 4–36% of ILD cases, whereas PR3-ANCA is less common, occurring in approximately 2–4% of patients [22]. Notably, ANCA seroconversion during the course of ILD has been reported in up to 11% of patients and is strongly associated with an increased risk of developing AAV [35]. Nevertheless, the clinical trajectories of patients with ILD and ANCA-positivity remain heterogeneous and unpredictable. Although ILD may precede the development of MPA, vasculitis may never manifest in a subset of these patients [33]. The risk of progression to MPA appears to be primarily linked to MPO-ANCA positivity rather than PR3-ANCA positivity [35,36]. However, one study indicated that PR3-ANCA positivity at the time of ILD diagnosis is an independent predictor of mortality in this patient population [35].

In a large retrospective cohort of 504 patients with IPF, baseline MPO-ANCA and PR3-ANCA positivity were observed in 4.0% and 3.2% of the patients, respectively. During a median 5-year follow-up period, seroconversion occurred in 5.7% and 5.3% of the MPO-ANCA- and PR3-ANCA-positive patients, respectively, with 25% of the MPO-ANCA-positive or seroconverted patients developing MPA [34]. In another study, approximately 40% of the ANCA-positive patients with IPF had pANCA at baseline, whereas 60% were seroconverted after a mean period of 32.4 months. In addition, approximately 50% of the patients developed vasculitis 30 months after IPF diagnosis [89].

The risk factors for the development of AAV in patients with ANCA-ILD are not fully understood. Some studies suggest that a high baseline RF level is associated with the onset of AAV, whereas a history of treatment with CS for ILD seems to be associated with a reduced risk of AAV development [89]. In a most recent study of 77 patients with rheumatoid arthritis (RA) and ANCA positivity, RA diagnosis preceded AAV in 59% of the patients [90]. Another report indicated that an increased percentage of eosinophils in BALF is correlated with a significantly increased risk of developing MPA [34]. Additionally, other studies have shown that RF positivity and an elevated erythrocyte sedimentation rate ≥ 40 mm/h are associated with an increased risk of conversion to MPO- and/or PR3-ANCA in patients with ILD [35].

The effect of ANCA on the clinical course of ILD remains unclear. Although the UIP pattern is the most common radiological finding, MPO-ANCA-positive patients frequently exhibit low attenuation areas [34], honeycombing, and irregularly shaped cysts [91]. Lung function decline appears to be similar between ANCA-positive and ANCA-negative patients with ILD [89]. However, MPO-positive patients may exhibit lower TL,co [34]. Although findings from some studies suggest that patients with ANCA-ILD show better survival than those with ANCA-negative ILD [89], the overall prognosis of ANCA-ILD remains poorer than that of AAV-ILD [92]. In addition, low predicted VC and higher ANCA titers are associated with worse outcomes [33,92], whereas non-UIP HRCT patterns are associated with a more favorable prognosis [92]. In contrast, other studies have shown comparable survival rates regardless of ANCA status [91,93]. However, differences in FVC improvement and hospitalization rates were reported in one of the studies [93]. Sakamoto et al. [94] assessed the risk of progressive pulmonary fibrosis (PPF) in a cohort of patients with ANCA-ILD, including 21 with MPA and 17 with isolated ANCA-ILD. During a follow-up period of at least 1 year, approximately 40% of the patients developed PPF, which occurred more frequently in patients with isolated ANCA-ILD than in those with MPA-ILD (53% vs. 27%, *p* = 0.126). In addition, PPF was associated with significantly worse survival, and its development was correlated with older age, elevated serum SP-D levels, and lower baseline %FVC.

Whether ANCA-positive ILD is a limited form of AAV or a distinct clinical entity remains debatable. Although ANCA positivity can coexist with ILD, such cases do not meet the classification criteria for interstitial pneumonia with autoimmune features (IPAF) [95]. Moreover, some studies have indicated that isolated ANCA-ILD differs clinically and serologically from IPAF [96]. In contrast, the 2022 ACR/EULAR criteria for MPA assigned point to ILD (+3) and MPO-ANCA positivity (+6), which made them meet the classification threshold. However, these criteria are intended for classification, not diagnosis, and should be used to categorize established AAV types (GPA, MPA, or EGPA) [97]. Nevertheless, some authors suggest that isolated ANCA-ILD may be a limited or lung-restricted form of MPA [16]. However, lung biopsies of patients with ANCA-ILD often show a UIP pattern without vasculitis features [82]. This discrepancy highlights the heterogeneity of ANCA-ILD and the need for prospective studies that integrate clinical, radiological, serological, and histopathological data to clarify its classification within or beyond the AAV spectrum.

Although routine ANCA screening of patients with ILD without signs of vasculitis has limited diagnostic value [98], growing evidence supports serial ANCA testing of patients with IPF [35,89]. However, the current ATS/ERS/JRS/ALAT guidelines do not recommend routine ANCA testing in this context [99]. Conversely, guidelines that are not applicable to AAV recommend ANCA testing for ILD diagnosis [58]. Due to the risk of AAV development, especially in patients with MPO-ANCA, patients with ANCA-ILD require careful, long-term monitoring for systemic vasculitis, including renal function and urinalysis, as up to 50% may develop glomerulonephritis after a median period of 3.2 years [16]. Although there is no consensus regarding the optimal frequency of ANCA monitoring, some authors suggest annual testing or repeat testing in response to clinical deterioration, while renal function and urinalysis should be performed every 6 months [89]. However, further research is required to improve the understanding and management of this patient subgroup.

## 8. Treatment Considerations

### 8.1. Choosing the Optimal Treatment Strategy

The optimal treatment for patients with ILD and ANCA positivity remains unclear and largely depends on the presence of clinically overt AAV and the radiological pattern of ILD. UIP typically indicates advanced fibrosis, whereas non-UIP patterns such as NSIP suggest increased inflammation and may respond better to IS therapy [18]. Histopathological findings, when available, can facilitate the personalization of treatment. Empirical clinical approaches suggest that AAV-ILD should be managed according to standard AAV protocols, whereas isolated ANCA-positive ILD with non-UIP patterns may benefit from IS [100]. In contrast, antifibrotic treatment may be more appropriate for UIP because IS therapy generally shows limited efficacy in UIP, as demonstrated in the PANTHER-IPF trial [100,101]. This approach aligns with guidelines that recommend antifibrotics for the treatment of progressive fibrosing (PF)-ILD and caution against the use of IS for UIP without systemic autoimmune features [99]. However, these approaches do not address the management of patients with AAV-ILD who develop progressive fibrosis despite receiving adequate IS. This highlights the need for refined treatment strategies and better diagnostic tools for individualized care in this heterogeneous patient group.

Although distinguishing the dominant component is critical for guiding ILD treatment, including in cases with ANCA-positivity, differentiating between inflammatory and fibrotic processes remains a major clinical challenge, as both frequently coexist and contribute to disease progression [11,102]. While HRCT is essential for disease evaluation, radiological patterns such as UIP or NSIP do not always correlate with the underlying histopathology. In patients with ANCA-ILD showing a UIP pattern, increased attenuation around areas of honeycombing may indicate active inflammation and help identify individuals who might benefit from IS therapy despite predominant fibrosis. [94]. Additional signs of active inflammation include systemic symptoms, elevated levels of inflammatory markers, and increasing ANCA titers [103]. Other biomarkers, such as serum Ca19-9 (associated with fibrotic activity) and CYFRA21-1 (reflecting inflammation), have also been proposed. However, their clinical utility remains to be established [104]. In addition, patient-related factors, including comorbidities, should be carefully considered when selecting a treatment strategy, as they may significantly affect both the efficacy and tolerability of therapy [11].

In the absence of validated composite endpoints for AAV-ILD and ANCA-ILD, various clinical, functional, radiologic, and laboratory parameters are used to monitor disease progression and treatment response. Table 1 provides an overview of these measures and their relative importance depending on the underlying phenotype.

### 8.2. When to Initiate Treatment

The optimal timing for initiation of therapy for patients with ILD and AAV, especially those in remission from other vasculitis manifestations or with isolated ANCA-positive ILD, remains unclear. Studies on PF-ILD beyond IPF suggest that disease progression should prompt the initiation of treatment, a concept likely applicable to AAV-ILD as well [105,106,107]. Data from prospective studies indicate that a UIP pattern and fibrosis affecting ≥20% of the lungs are independent predictors of progression in non-IPF fibrotic ILD [108]. Specific progression criteria for AAV-ILD have been proposed based on these findings and include a ≥10% relative decline in FVC, a ≥15% decline in TL,co, a >50 m decrease in 6-MWT within 12 months, or radiographic worsening on HRCT—each of which may justify treatment initiation [32]. Interestingly, these criteria emphasize the importance of timely therapeutic intervention guided by disease trajectory rather than baseline severity alone. Quantitative CT biomarkers have shown promise in monitoring progression independent of the GAP severity index, imaging patterns, or circulating biomarkers, facilitating earlier treatment adjustments and supporting decisions regarding integration of supportive or palliative care [109]. However, careful monitoring and strategy “watch and wait” may be appropriate for asymptomatic patients with stable or nonprogressive ILD [22,92].

Due to the clinical heterogeneity of ILD in ANCA-positive cases, management should be personalized and multidisciplinary. Collaboration among pulmonologists, radiologists, rheumatologists, and, when applicable, pathologists is essential for defining clinical phenotypes and guiding treatment [15]. Similarly, close cooperation between vasculitis experts and ILD specialists is crucial for optimizing the outcomes of AAV-ILD through an integrated and individualized treatment approach [15].

### 8.3. Current Treatment Approaches

#### 8.3.1. Standard Treatment

Despite its clinical importance, there is no standardized treatment for AAV-ILD due to the lack of data from controlled trials [56], and current management strategies are largely based on systemic AAV therapies. Induction of remission typically involves the administration of CS combined with cyclophosphamide (CYC) or, more commonly, rituximab (RTX) [56,110]. RTX is preferred for maintenance, showing superior efficacy to conventional IS after induction [111]. However, agents such as methotrexate (MTX), azathioprine (AZA), and mycophenolate mofetil (MMF) are suitable alternatives. Notably, MMF is associated with a higher risk of relapse [112]. However, recent meta-analyses have suggested its benefits in cases with renal involvement [113]. Avacopan, a C5a receptor antagonist, is a steroid-sparing agent approved for the treatment of AAV [114]. However, its effects on ILD have not been studied. Since no prospective study has been conducted to specifically assess the effect of IS therapy on ILD in AAV, the current understanding is mainly based on retrospective analyses, which have produced conflicting results.

Evidence on the efficacy of IS therapy in AAV-ILD remains mixed, with several studies supporting its benefit, while others report limited or no effect—particularly in patients with a UIP pattern.

A study by the French Vasculitis Study Group, which included 49 patients with AAV-associated pulmonary fibrosis, demonstrated that induction therapy with CYC improved outcomes, whereas CS monotherapy increased mortality by 1.7-fold. Interestingly, the HRCT pattern at baseline was not found to be a prognostic factor [42]. Similar findings were reported in a Chinese cohort of 155 patients with AAV (including 112 with ILD), where IS therapy used for induction was associated with better outcomes [84]. A meta-analysis of 654 patients with AAV-ILD further confirmed that induction with IS therapy significantly reduced the risk of mortality [87]. In a UK retrospective study of 69 patients with ANCA-positive ILD, lung function declined without treatment but improved over a 12-month period in those receiving IS therapy [69]. Additionally, in a cohort of 97 patients with MPA (38% with ILD), CS use independently correlated with improved survival among patients with ILD [115].

In contrast, other studies suggest more nuanced or even unfavorable effects of IS in certain subgroups. Maillet et al. [54], in a cohort of 62 patients with AAV-ILD, found no significant benefit from IS treatment and proposed that conventional therapies might even worsen outcomes in patients with a UIP pattern. This observation is consistent with a meta-analysis indicating that IS-based induction therapy did not improve survival in AAV-ILD overall—particularly in those with a UIP pattern—whereas better outcomes were observed in patients with non-UIP ILD, likely reflecting a more favorable response to CS and IS therapy [18].

Although RTX is widely used for the treatment of AAV, its effect on ILD remains unclear due to a lack of evidence from dedicated studies. Some reports suggest a trend toward better preservation of lung function. However, no statistically significant benefit of RTX-based regimens over non-RTX strategies has been shown in patients with AAV-ILD, especially those with a UIP pattern [68].

Regarding ANCA-positive ILD, available data are similarly limited. Evidence suggests that combination therapy with CS and IS agents is independently associated with improved survival [91], whereas CS monotherapy appears to offer limited benefit [92]. However, contrasting results were reported in a comparative study of isolated ANCA-ILD and ILDs classified as IPAF. While CS and IS therapy improved pulmonary function in patients with IPAF, those with ANCA-ILD often showed continued lung function decline after 1 year of treatment and had significantly higher all-cause mortality [96].

As a result, treatment strategies for patients with ANCA-ILD without systemic vasculitis are largely extrapolated from therapeutic approaches used in other ILDs. For patients with predominant UIP patterns, current practice follows data supporting the use of antifibrotic agents in PF-ILD (non-IPF) [105,116], with additional guidance derived from IPF studies [99]. In contrast, for non-UIP patterns, treatment is often based on existing evidence for connective tissue disease (CTD)-associated ILDs, which favors the use of IS therapies such as CYC or MMF, including B-cell-depleting agents [117,118,119]. Much of this evidence originates from studies on systemic sclerosis (SSc)-associated ILD, in which up to 75% of patients are reported to present with an NSIP pattern—considered primarily inflammatory in nature [120].

Although primarily focused on CTD-ILDs, emerging data on RTX may offer insights relevant to the treatment of ANCA-ILDs. Overall, findings from studies tend to favor RTX over other therapies in CTD-ILDs [119]. A meta-analysis reported modest improvements in %FVC and %TL,co (approximately 4–5%) after treatment with RTX, compared to stabilization observed with alternative therapies [121]. However, in the RECITAL trial, although patients treated with RTX experienced fewer adverse events, no significant difference in efficacy was observed between RTX and CYC; the mean increase in FVC was 97 mL vs. 99 mL, respectively [122]. In turn, in the EvER-ILD trial, the combination of RTX and MMF was superior to MMF alone in treating NSIP-pattern ILD, while the incidence of viral infections remained comparable between the two treatment groups [123]. Results from the ongoing EvER-ILD2 trial, which investigates PF-ILD with inflammatory features, are expected in 2026 (NCT05596786) [124].

Recently, the American College of Rheumatology and the American College of Chest Physicians published joint guidelines on the treatment of ILD in patients with systemic autoimmune rheumatic diseases, which may also apply to ANCA-ILD [125]. Developed mainly by rheumatologists, the recommendations prioritize MMF as a first-line treatment for SSc-ILD, with tocilizumab (targeting interleukin 6) and RTX as alternatives, and advise against long-term CS use. Regarding other CTD-ILDs, MMF, AZA, and RTX are preferred, with CS recommended for short-term use. CYC and Janus kinase (JAK) inhibitors are second-line treatment options. An antifibrotic agent (nintedanib) may be added in cases of PF-ILDs that are unresponsive to initial therapy [125].

Since many of these recommendations are conditional, treatment should be tailored to individual patients.

#### 8.3.2. Antifibrotic Therapy

Owing to the complexity of managing fibrotic lung diseases, antifibrotic agents such as pirfenidone and nintedanib are increasingly being incorporated into treatment strategies. These drugs have been shown to slow disease progression [126,127], reduce acute exacerbations [128], and improve survival in patients with IPF [129], supporting their potential role in the treatment of a wider range of fibrosing ILDs, including ILD in patients with AAV and ANCA positivity. These extended roles of antifibrotic agents are reinforced by data from studies on other autoimmune diseases. In the SENSCIS trial, nintedanib significantly slowed FVC decline in patients with SSc-ILD (−52.4 vs. −93.3 mL/year; *p* = 0.04) [130]. Similarly, the INBUILD trial confirmed a decrease in FVC decline in patients with diverse PF-ILDs, including autoimmune-related cases [105]. In turn, findings from the RELIEF trial support the efficacy of pirfenidone in the treatment of fibrosing ILDs unresponsive to standard therapy. However, early termination of the therapy limits interpretation of the findings [131]. Additional data from other studies suggest the benefits of pirfenidone in the treatment of RA-ILD despite the composite endpoint not being met in a study. In addition, some studies indicate that pirfenidone shows beneficial effects in fibrosing hypersensitivity pneumonitis (HP), improving progression-free survival despite no significant difference in FVC [132,133].

Growing evidence supports the potential benefits of combining antifibrotic and IS therapies for the treatment of autoimmune-related ILDs. A retrospective study conducted by Ushio et al. [134] demonstrated improved lung function in patients with CTD-ILDs treated with nintedanib and IS therapy compared to those treated with nintedanib alone. However, the optimal timing and strategy for such combination therapy remain to be determined. Notably, a prospective clinical trial (NCT04928586) on the efficacy of pirfenidone combined with IS agents for the treatment of CTD-ILD is currently underway. 

Interest in dual antifibrotic therapy has also grown. Despite pirfenidone and nintedanib having overlapping side-effect profiles, primarily gastrointestinal [135], a recent meta-analysis showed that the combination of both drugs is generally well tolerated, with a safety profile similar to that of monotherapy and a potential additional benefit in slowing FVC decline [136].

As AAV-ILD shares key features with other fibrosing ILDs, interest in antifibrotic therapy for AAV-ILD is increasing. However, prospective studies on this topic are lacking. The only pilot trial (PIRFENIVAS) on pirfenidone for the treatment of MPO-ANCA-positive ILD (with or without AAV) was terminated early because of poor enrollment (NCT03385668). Due to the recent approval of antifibrotics for PF-ILD regardless of etiology, their use for the treatment of AAV-ILD or isolated ANCA-ILD appears reasonable. However, further validation in future trials is warranted. Clinically, antifibrotic therapy may be considered for select patients, particularly those with a progressive phenotype and/or a predominant UIP pattern, especially when IS therapy fails to control the disease. Until stronger evidence emerges, such decisions should be based on multidisciplinary evaluations and individualized risk–benefit assessments.

**Figure 4:**
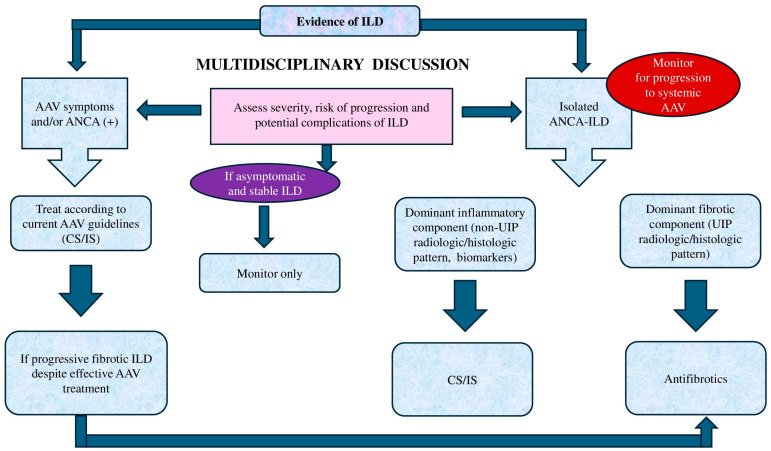
Proposed management algorithm for patients with AAV-ILD and isolated ANCA-ILD. In patients with symptomatic AAV, treatment should be administered in accordance with current AAV guidelines. In patients with isolated ANCA-ILD, therapeutic decisions should depend on the predominant ILD component—either inflammatory or fibrotic. IS therapy is recommended for patients with a dominant inflammatory pattern, whereas antifibrotic treatment should be considered when fibrosis prevails. Antifibrotics may also be considered for patients with PF-ILD in AAV despite effective control of systemic AAV manifestations. Treatment may not be necessary if ILD is mild and non-progressive; a “watch and wait” approach may be most appropriate in such scenarios. Therefore, at the time of ILD diagnosis, assessment of disease severity, risk of progression, and potential complications is essential. Therapeutic decisions should ideally be based on multidisciplinary discussions.

The treatment strategy for ILD in patients with AAV and/or ANCA positivity is outlined in Figure 4.

### 8.4. Emerging Therapies and Future Directions

Several clinical trials on novel therapeutic approaches for AAV are ongoing. However, none are specifically focused on ILD (Table 2). Research on ILD, particularly IPF, has led to the development of innovative therapies, including targeted immunomodulators and antifibrotic agents (Table 3). Many of these therapies are currently being evaluated for the treatment of other PF-ILDs (Table 4). Due to the predominantly fibrotic phenotype observed in AAV-ILD and ANCA-ILD, and the mechanistic overlap with other immune-mediated pulmonary disorders, therapies designed for IPF or PPF may ultimately hold therapeutic potential for patients with AAV-ILD or ANCA-ILD.

Among the emerging therapies, admilparant and nerandomilast have shown particular promise in the treatment of pulmonary fibrosis. Admilparant (BMS-986278), a selective lysophosphatidic acid receptor 1 (LPAR1) antagonist involved in fibrotic signaling pathways [137], was assessed in a phase 2 trial that included patients with IPF and PPF. The drug significantly reduced lung function decline over 26 weeks compared to the placebo (IPF: −1.2% vs. −2.7%; PPF: −1.1% vs. −4.3%) and was well tolerated [138]. Phase 3 studies that include patients with IPF and PPF are currently ongoing (ALOF-IPF and ALOF-PPF). Nerandomilast (BI-1015550), an oral phosphodiesterase (PDE) 4B inhibitor involved in inflammatory and fibrotic signaling [139], also showed efficacy in a phase 2 trial of patients with IPF, both as monotherapy and in combination with antifibrotic agents [140]. This led to two phase 3 trials, FIBRONEER-IPF and FIBRONEER-PPF, which were completed in December 2024 and April 2025, respectively [141,142]. Results from the FIBRONEER-IPF trial have been recently published, demonstrating that treatment with nerandomilast resulted in a smaller decline in FVC over 52 weeks compared to placebo (−114.7 mL vs. −183.5 mL) [143]. Preliminary findings from the FIBRONEER-PPF trial also indicate promising efficacy and good tolerability. A long-term extension study (FIBRONEER-ON; NCT06238622) is currently underway.

Next, inhaled drug delivery to reduce systemic toxicity and improve pulmonary drug targeting is currently being explored. In the phase 1b ATLAS trial, inhaled pirfenidone (AP01) demonstrated favorable tolerability and markedly lower systemic exposure than oral formulations in patients with IPF [144]. Its efficacy in the treatment of PPF is currently being assessed in the APO1-007 (MIST) trial, with endpoints including FVC decline and cough burden [145]. Although the available data on inhaled nintedanib are preclinical [146,147], nanoparticle-based formulations have shown antifibrotic potential in vitro, supporting further evaluation of this route of administration [148].

Inhaled treprostinil, an analog of prostacyclin, is also being actively investigated for the treatment of ILDs. In the INCREASE trial, which included 326 patients with ILD and pulmonary hypertension, inhaled treprostinil significantly improved exercise capacity compared to placebo, with a mean 6-MWT gain of 21.08 m versus a decline of −10.04 m [149]. Based on these results, phase 3 studies on the use of inhaled treprostinil for the treatment of IPF (TETON, TETON-2) [150] and PPF (TETON-PPF) [151] have been conducted, with the primary focus being FVC changes at 52 weeks. In addition, several novel inhaled agents, including TD139 (galectin-3 inhibitor), TRK250 (TGF-β1 modulator), DMF (Nrf2 activator), and CL27c (pan-PI3K inhibitor), are currently in early development. However, most of the studies are in phase 1 or preclinical stages [152].

Another promising treatment approach is targeting the JAK/signal transducer and activator of transcription (STAT) pathway, which contributes to fibrotic signaling. Preclinical data suggest that inhibition of this pathway may reduce inflammation and the progression of fibrosis [153]. Phase 2 trials on evaluation of the STAT3 inhibitor TTI-101 (NCT05671835) and the JAK1–3 inhibitor jakitinib (NCT04312594) are ongoing, with experimental evidence supporting dual JAK/STAT blockade in fibrotic ILDs [154]. JAK inhibitors are increasingly being used for the treatment of refractory CTDs, and their potential role in the treatment of ILD is being explored, particularly in RA-ILD, where the UIP pattern resembles that observed in AAV-ILD. Results of retrospective studies suggest that this approach leads to short-term stabilization of lung function and HRCT findings [155,156]. However, treatment discontinuation due to infection was frequently reported in these studies [156]. A meta-analysis on the use of JAK inhibitors for the treatment of RA-ILD confirmed modest but significant improvements in %FVC (+2.07%, *p* = 0.007) and %TL,co (+3.12%, *p* < 0.001) after treatment [157]. Ongoing trials, such as PULMORA (NCT04311567) and RAILDTo (NCT05246293), are being conducted to evaluate the efficacy of tofacitinib for the treatment of RA-ILD.

Tofacitinib has shown benefits in the treatment of small cohorts of patients with refractory GPA, particularly those with granulomatous involvement of the airways [158,159]. Notably, ILD was not addressed in these studies. A phase 4 trial on the comparison of tofacitinib and MTX for maintenance of remission in patients with GPA (NCT04944524) has been initiated. However, its current status remains unclear.

Notably, apart from the prematurely terminated PIRFENIVAS study, no clinical trial to date has specifically targeted AAV-ILD. However, several investigational therapies emerging from the broader field of fibrosing ILDs—including those studied in CTD-ILD—may hold promise in this setting.

Emerging approaches such as CAR-T cell therapy targeting B-cell maturation antigens (NCT06277427, NCT06462144, NCT06590545) further expand the spectrum of potential future interventions for refractory AAV. Although current trials do not specifically address AAV-ILD, these innovative strategies could ultimately prove beneficial in selected cases with persistent inflammatory activity unresponsive to conventional treatment.

Similarly, regenerative therapies with allogeneic mesenchymal stem cells (NCT06574581) or B-cell activating factor inhibition with belimumab (NCT06572384) reflect the ongoing search for novel immunomodulatory approaches beyond standard IS therapy. Due to the mechanistic overlap among autoimmune-driven fibrosing lung diseases, such interventions may potentially be applicable to AAV-ILD, particularly in refractory disease.

Nonetheless, robust clinical data will be required to determine their safety and efficacy in the context of ANCA-associated ILD.

#### Precision Medicine

Precision medicine is gaining importance in the management of fibrosing ILDs. Its aim is to improve patient stratification and enable personalized therapy by integrating behavioral, environmental, molecular, epigenetic, and genetic profiling [160].

A post-hoc analysis of the PANTHER trial identified a genotype–treatment interaction in patients with IPF: those with the TOLLIP rs3750920 TT genotype appeared to benefit from acetylcysteine despite its lack of efficacy in the overall trial population [101]. This led to the PRECISIONS phase 3 trial, which enrolled 200 IPF patients with this genotype to receive oral acetylcysteine or placebo for 24 months; results are expected in 2026. This genotype-guided approach may mark a turning point in developing personalized treatments for fibrotic lung disease.

Telomere dysfunction is another emerging target. Shortened telomeres are associated with poor outcomes, including higher mortality and post-transplant complications, particularly in IPF and fibrotic HP [161,162]. Trials such as NCT03312400 and TELO-SCOPE are evaluating agents like danazol in patients with telomere-related disorders, including pulmonary fibrosis.

Finally, the PRECISION-ILD study (NCT05998512) is a large observational trial including 1000 patients with fibrotic ILD (≥5% fibrosis on CT), designed to explore genetic and environmental factors affecting disease progression and treatment response. It also evaluates the feasibility of implementing biomarker-based, predictive, preventive, personalized, and participatory (P4) medicine. Completion is expected in December 2025.

In AAV, genetic research is advancing, although ILD-specific data remain scarce. Certain variants associated with pulmonary fibrosis—such as the MUC5B promoter polymorphism, and TERT and DSP mutations—may help identify patients at risk of progressive fibrosis [52,53]. PR3-AAV and MPO-AAV differ in relapse risk and treatment response, reflecting distinct genetic backgrounds. Genome-wide studies have linked PR3-AAV to HLA-DPB1, HLA-DPA1, SERPINA1, and PRTN3, and MPO-AAV to HLA-DQA2 and DQB1 [6]. These findings support the notion that ANCA-defined AAV subtypes are biologically distinct.

Treatment response appears to align with the genotype: PR3-AAV responds better to RTX than to CYC/AZA, while efficacy is similar for both regimens in MPO-AAV [60]. Additionally, the PRTN3-Val119Ile polymorphism has been linked to relapse in PR3-AAV [163], and specific HLA class II variants (DRB109:01, DQA103:02, and DQB1*03:03) are associated with relapse in MPO-AAV [164].

Although current applications of precision medicine in AAV-ILD remain theoretical, emerging genetic and molecular insights may ultimately support biomarker-driven, stratified treatment approaches.

These findings have not yet translated into routine clinical practice but provide a foundation for future individualized strategies. Still, genetic studies focused specifically on ILD in AAV remain limited, underscoring the need for further research.

### 8.5. Supportive Care

Supportive care is a key component in the management of advanced ILD, regardless of etiology, and remains essential in AAV-ILD and ANCA-positive ILD. It includes oxygen therapy, pulmonary rehabilitation, infection prevention, and symptom management—particularly of chronic cough, which significantly impacts quality of life.

Home oxygen therapy is often needed to alleviate symptoms, correct hypoxemia, and prevent complications such as pulmonary hypertension. In a large cohort of patients with PF-ILD, nearly 40% required oxygen within 5 years of diagnosis [165]. Initiation is recommended in cases of resting hypoxemia (PaO_2_ < 55 mmHg or SpO_2_ < 89%), or PaO_2_ < 60 mmHg with cor pulmonale or polycythemia [166]. Ambulatory oxygen improves quality of life [167], and the ongoing OXYODE trial is evaluating optimal delivery strategies.

Pulmonary rehabilitation improves dyspnea, fatigue, and exercise capacity across ILD subtypes [168,169]. However, its effects may decline over time, highlighting the need for sustained programs. A current trial (NCT06527612) is assessing long-term outcomes of rehabilitation in PF-ILD, with results expected in 2026.

Preventing infections is particularly important in AAV-ILD, especially during IS treatment. Prophylaxis with trimethoprim/sulfamethoxazole against *Pneumocystis jirovecii* is recommended [56], along with vaccinations against pneumococcus, influenza, COVID-19, and RSV. The PNEUMOVAS trial showed enhanced vaccine responses in RTX-treated patients receiving intensified PCV13/PPSV23 schedules, although ILD-specific data are lacking [170].

Management of chronic cough—a common and burdensome symptom—is also essential. Its pathogenesis is multifactorial and may involve airway remodeling, neural sensitization, and comorbidities such as GERD [171]. Pharmacologic options include neuromodulators, opioids, and antireflux therapy. Among antifibrotics, nintedanib has been associated with cough reduction, unlike pirfenidone [122,130,172,173]. New strategies such as P2X3 receptor antagonists and airway hydration are under investigation [171].

### 8.6. Lung Transplantation

Lung transplantation remains the definitive option for advanced ILD. However, patients with AAV-ILD are often poor candidates because of their age, the presence of comorbidities, and systemic involvement [174]. Guidelines recommend a referral when FVC < 80%, TL,co < 40%, or in cases of rapid progression (e.g., ≥10% FVC or ≥15% TL,co decline within 2 years) [174]. In cases of autoimmune ILD, including AAV-ILD or ANCA-positive cases, early referral is advised because of potential extrapulmonary involvement. Individualized risk assessment is essential because treatment outcomes vary [175].

## 9. Conclusions

ILD in patients with AAV presents a diagnostic and therapeutic challenge. The distinction between isolated ANCA-ILD and AAV-ILD remains unclear, raising the question of whether ANCA-ILD is an early or limited form of systemic vasculitis. Despite advancements in AAV treatment, ILD remains a major cause of morbidity and mortality, particularly in patients with progressive fibrosing disease. Although IS therapy remains central to treatment, evidence suggests that antifibrotic agents may play a beneficial role. However, the lack of clinical trials specifically focused on AAV-ILD highlights the urgent need for further research. A personalized treatment approach that integrates biomarker analysis, radiographic assessment, and risk stratification is crucial for optimizing patient outcomes. This will allow for tailored management and adjustment of therapies based on individual disease characteristics, genetic factors, and previous treatment responses. Future research should be focused on the complex interplay between inflammation and fibrosis, as understanding these mechanisms may reveal novel therapeutic targets for AAV-ILD. Ultimately, expanding our knowledge of the genetic, immunological, and fibrotic pathways involved in AAV-ILD and isolated ANCA-ILD will lead to more effective and individualized treatments, thereby improving the prognosis and quality of life of patients.

## Figures and Tables

**Table 1:** Clinical parameters used to assess disease progression and treatment response in AAV-ILD and ANCA-ILD.

Parameter	Fibrotic-Predominant Disease	Inflammatory-Predominant Disease	Clinical Priority/Comments
FVC decline	High	Moderate	Main functional parameter used in most studies
TL,co decline	High	Moderate	Sensitive to gas exchange impairment
HRCT progression	High	High	Useful in both phenotypes; qualitative and quantitative assessment
6-MWT	Moderate	Moderate	Reflects functional status, influenced by comorbidities
CRP/ESR	Low	High	Useful markers of systemic inflammation
ANCA titers	Low to moderate	High	Dynamic changes may have clinical relevance, though not fully validated
Renal function/ urinalysis	High	High	Critical for detecting systemic involvement (e.g., glomerulonephritis)

Abbreviations: AAV-ILD, interstitial lung disease associated with anti-neutrophil cytoplasmic antibody-associated vasculitis; ANCA-ILD, interstitial lung disease with anti-neutrophil cytoplasmic antibody positivity; FVC, forced vital capacity; TL,co, transfer factor of the lungs for carbon monoxide (DLCO); HRCT, high-resolution computed tomography; 6-MWT, 6-min walk test; CRP, C-reactive protein; ESR, erythrocyte sedimentation rate.

**Table 2:** Ongoing clinical trials in AAV (primarily focused on GPA and MPA).

Study Name (ClinicalTrials.gov ID)	Studied Drug	Estimated Enrolment	Primary Outcome	Phase	Status	Estimated Completion Date
ENDURRANCE-1 (NCT03942887)	Combination of RTX and CYC vs RTX monotherapy	GPA MPA (N = 100)	Number of RTX-tailored infusions	3	Active, recruiting	April, 2025
HAVEN (NCT04316494)	Hydroxychloroquine added to background treatment	GPA MPA EGPA (N = 76)	Patients (%) with either: uncontrolled AAV (BVAS > 3), or controlled AAV (BVAS ≤ 3) but requiring > 7.5 mg/day of prednisolone (any reason) at week 12	4	Active, not recruiting	May, 2025
SATELITE (NCT04871191)	RTX + DMARD vs RTX + tocilizumab vs RTX + abatacept in induction of remission	GPA (N = 42)	Patients (%) with response or remission at week 12	3	Not yet recruiting	January, 2029
NCT05376319	Obinutuzumab (anti-CD20)	PR3-AAV (N = 6)	Patients achieving CR and ANCA seronegativity at 6 months	2	Early terminated (due to sponsor funding withdrawal)	
AVACOSTAR (NCT05897684)	Avacopan vs. CYC- or RTX-based induction regimens for severe AAV	AAV (N = 500)	The incidence of MESIs in patients commencing Avacopan	Observational	Recruiting	December, 2030
NCT05197842	BDB-001 (anti-C5RA1 monoclonal antibody activating the toll-like receptor)	AAV (N = 100)	Proportion of patients achieving CR or PR at 12 weeks	1/2	Recruiting	March, 2025
IDEAL (NCT06590545)	anti-CD19 CAR-T cell antibodies	AAV refractory, with ANCA-IgG-positivity (N = 8)	Number of subjects experiencing CRS up to 4 weeks ANCA seroconversion rate at 24 weeks AEs and SAEs up to 52 weeks	1/2	Not yet recruiting	July, 2027
NCT06462144	IMPT-514 (anti-CD19/CD20 CAR-T cell antibodies)	AAV SLE IIM (N = 36)	Incidence of DLTs, SAEs, and TEAEs up to 28 days post-infusion	1	Recruiting	October, 2026
Ntrust-2 (NCT06733935)	NKX019 (allogeneic CAR-NK cell targeting CD19)	Immune-mediated diseases including AAV (N = 72)	Incidence of DLTs and TEAEs	1	Recruiting	October, 2028
TTCAAVREM (NCT05962840)	Telitacicept (targeting Blys and APRIL) + RTX in induction vs Telitacicept alone in maintenance	AAV (N = 40)	Time to first relapse during 24-month follow-up in two groups	4	Recruiting	December, 2026
NCT06277427	PRG-1801 (CAR-T cell targeting B-cell maturation antigen)	AAV (N = 24)	AEs incidence at 24 months after infusion Types and incidence of DLTs at 28 days and 3 months after infusion	Not applicable	Recruiting	January, 2027
NCT06388941	Iptacopan (factor B inhibitor) added to standard of care therapy	GPA (N = 78)	Sustained remission through week 48 (CR at week 24 without major relapse)	2	Recruiting	October, 2027
NCT06226662	NM8074 (antibody inhibiting Bb component of complement) added to standard of care therapy	GPA MPA RLV (N = 12)	Proportion of subjects with disease response at day 85	2	Recruiting	September, 2027
NCT06196905	MT-2990 (monoclonal antibody targeting IL-33)	GPA MPA EGPA (N = 10)	No primary endpoint; exploratory analyses: change in BVAS/VDI and FVC up to 24 weeks	1	Recruiting	February, 2026

Abbreviations: RTX, rituximab; CYC, cyclophosphamide; GPA, granulomatosis with polyangiitis; MPA, microscopic polyangiitis; EGPA, eosinophilic granulomatosis with polyangiitis; AAV, anti-neutrophil cytoplasmic antibody associated vasculitis; BVAS, Birmingham Vasculitis Activity Score; DMARD, disease-modifying antirheumatic drugs; CR, complete remission; PR, partial remission; PR3, proteinase 3; MESIs, medical events of special interest; CAR, chimeric antigen receptor; ANCA, anti-neutrophil cytoplasmic antibody; CRS, cytokine release syndrome; IgG, immunoglobulin G; AEs, adverse events; SAEs, severe adverse events; SLE, systemic lupus erythematosus; IIM, idiopathic inflammatory myopathy; DLTs, dose limiting toxicities; TEAEs, treatment emergent adverse events; NK, natural killers; Blys, B lymphocyte stimulator; APRIL, A proliferation—inducing ligand; RLV, renal limited vasculitis; IL-33, interleukin 33; VDI, Vasculitis Damage Index; FVC, forced vital capacity.

**Table 3:** Ongoing clinical trials for the treatment of IPF.

Study Name (ClinicalTrials.gov ID)	Studied Molecule (Mechanism)	Estimated Enrolment	Primary Outcome	Phase	Status	Estimated Completion Date
REVERT-IPF (NCT05671835)	TTI-101 (STAT3 inhibitor)	N = 75	Participants with AEs at 16 weeks	2	Recruiting	July, 2025
NCT05483907	BBT-877 (selective autotaxin inhibitor)	N = 129	FVC change from baseline at 24 weeks	2	Active, not recruiting	February, 2025
NCT05571059	Ifetroban (selective thromboxane antagonist)	N = 128	FVC change from baseline at 12 months	2	Recruiting	January, 2026
MAXPIRe (NCT06132256)	Axatilimab (CSF-1R targeting antibody)	N = 135	Annualized FVC decline (morning pre-dose) at 26 weeks	2	Recruiting	June, 2025
BEACON-IPF (NCT06097260)	Bexotegrast PLN-74809 (αVβ1 and αVβ6 integrin inhibitor)	N = 360	Change from baseline in absolute FVC at week 52	2	Active, not recruiting	September, 2025
ALOFT-IPF (NCT06003426)	Admilparant BMS-986278 (LPA1 antagonist)	N = 1185	Participants with SSEs and FVC change from baseline up to week 52	3	Recruiting	October, 2026
RIN-PF-301 (NCT04708782)	Inhaled treprostinil (prostacyclin analog)	N = 576	Absolute FVC change from baseline to week 52	3	Active, not yet recruiting	June, 2025
RIN-PF-303 (NCT05255991)	Inhaled treprostinil (prostacyclin analog)	N = 597	FVC change (absolute) from baseline to week 52	3	Active, not yet recruiting	July, 2025
NCT05785624	Vixarelimab (IL-31 inhibitor)	IPF SSc-ILD N = 320	FVC change (absolute) from baseline to week 52	2	Recruiting	August, 2027
NCT05389215	DWN12088 (prolyl-tRNA synthetase inhibitor)	N = 102	FVC decline rate at week 24 and incidence of AEs	2	Recruiting	December, 2025
NCT05195918	EGCG (green tea extract reducing mRNA levels and collagen I accumulation)	N = 50	Participants with AEs up to 12 weeks	1	Recruiting	April, 2026
PRECRSIONS (NCT04300920)	N-acetylocysteine (antioxidant)	IPF patients with TOLLIP rs3750920 TT genotype N = 202	Time to composite endpoint: 10% FVC decline, respiratory hospitalization, lung transplant, or death (24 months)	3	Active, not recruiting	February, 2026
TIPAL (NCT04965298)	Lansoprazole (proton pump inhibitor)	N = 298	Absolute %pred FVC change at 12 months post-randomisation	3	Recruiting	February, 2025
NCT03312400	Danazol (androgen hormone)	Telomere related diseases, including IPF N = 40	Telomere attrition reduction at 6 months and pulmonary function progression at 6 and 12 mths (secondary outcome)	2	Recruiting	October, 2027
TELO-SCOPE (NCT04638517)	Danazol (androgen hormone)	IPF in children and adults N = 50	Absolute telomere length change from baseline at 12 months and FVC and DLCO change at 6 and 12 months (secondary outcome)	2	Recruiting	June, 2025
WHISTLE-PF (NCT06422884)	ENV-101 (Hedgehog inhibitor)	N = 200	Rate of %ppFVC change vs. placebo at 24 weeks	2	Recruiting	June, 2026
NCT05515627	Atezolizumab (PD-L1 targeting antibody)	N = 24	Incidence of treatment-emergent AEs over 24 weeks	1	Recruiting	April, 2026
NCT06125327	Sufenidone SC1011 (antifibrotic)	N = 210	Annual FVC decline rate at 52 weeks	2/3	Recruiting	December, 2027
TRANFORM (NCT06317285)	GSK3915393 (TG-2 inhibitor)	N = 150	Absolute FVC change from baseline at week 26	2	Recruiting	March, 2026
ELEVATE (NCT05321420)	Duepirfenidone LYT-100 (antifibrotic—selective deuterated pirfenidone)	N = 240	FVC decline rate at 26 weeks	2	Active, not recruiting	December, 2025
NCT05537025	Inhaled ARO-MMP7 (MMP7 expression reduction)	IPF and healthy volunteers N = 97	Participants with treatment-emergent AEs over time	1/2	Recruiting	March, 2025

Abbreviations: IPF, idiopathic pulmonary fibrosis; STAT3, signal transducer and activator of transcription 3; AEs, adverse events; (pp)FVC, (percent predicted) forced vital capacity; CSF-1R, colony stimulating factor 1 receptor; IL-31, interleukin 31; SSc-ILD, systemic sclerosis-associated interstitial lung disease; tRNA, transfer rybonucleic acid; mRNA, messenger ribonucleic acid; TOLLIP, toll interacting protein; DLCO, diffusing-capacity for carbonmonoxide; PD-L1, programmed death receptor 1; SSEs, spontaneous syncopal events; LPA1, lysophosphatilic acid receptor 1; TG-2, transglutaminase 2; EGCG, epigallocatechin-3-gallate; MMP7, matrix of metalloproteinase 7.

**Table 4:** Ongoing clinical trials in fibrotic ILDs (other than IPF).

Study Name (ClinicalTrials.gov ID)	Studied Molecule (Mechanism)	Patient Cohort Estimated Enrolment	Primary Outcome	Phase	Status	Estimated Completion Date
EvER-ILD2 (NCT05596786)	RTX (anti-CD20 antibody)	PF-ILD with inflammatory component (N = 126)	FVC change at 6 months	3	Recruiting	July, 2026
EvER-ILD3 (NCT06549231)	RTX combined with MMF (T/B cell proliferation inhibitor) vs MMF alone	SSc-ILD N = 102	Change in ppFVC from baseline to week 24	3	Not yet recruiting	November, 2028
BEconneCTD-ILD (NCT06572384)	Belimumab (BLys inhibitor)	CTD-ILD N = 440	Absolute change in FVC from baseline at week 52	3	Recruiting	December, 2028
RAILDTo (NCT05246293)	Tofacitinib (JAK1-3 kinase inhibitor)	RA-ILD N = 60	Incidence and severity of AEs	2	Unknown	March, 2025
FIBRONEER-ON (NCT06238622)	Nerandomilast BI-1015550	PF-ILD/IPF N = 1700	Occurrence of AEs up to 99 weeks and 3 days	3	Recruiting	May, 2027
NCT06806592	Nerandomilast BI-1015550	RA-ILD N = 400	Absolute change in QILD score (%) at week 26	3	Not yet recruiting	March, 2027
NCT06440746	Olokizumab (anti-IL6 antibody)	PF-ILD N = 138	FVC decline rate over 48 weeks	2/3	Recruiting	December, 2028
NCT05828953	Anlotinib (TK inhibitor)	PF-ILD IPF N = 30	Absolute change in FVC at week 52	2/3	Recruiting	July, 2025
SOLIS (NCT06325696)	Hymecromone H01 (hyaluronan synthesis inhibitor)	ILD Lung fibrosis N = 37	Serum HA levels over 72 weeks and change in clinical and functional measures (secondary outcome)	2	Recruiting	December, 2027
NCT06329401	Inhaled pirfenidone APO1 (antifibrotic)	PF-ILD N = 300	Change in FVC from baseline at week 52	2	Recruiting	April, 2026
NCT06574581	AD-MSC (adipose tissue-derived mesenchymal stromal cells)	CTD-ILD N = 16	Safety profile over 48 weeks	1/2	Recruiting	May, 2026
NCT06825169	NCR101 (human induced pluripotent stem cell-derived mesenchymal stromal cells	Variable ILDs including IPF, HP, CTD-ILD N = 30	Incidence of AEs and SAEs	1/2	Not yet recruiting	September, 2028
ALOFT-PPF (NCT06025578)	Admilparant BMS-986278 (LPA receptor antagonist)	PF-ILD N = 1092	Number of participants with SSEs at 4 weeks and absolute change in FVC at week 52	3	Recruiting	December, 2027
TETON-PPF (NCT05943535)	Inhaled treprostnil (prostacyclin analog)	PF-ILD N = 698	Change in absolute FVC from baseline to week 52	3	Recruiting	November, 2027
NCT05649722	Treprostinil palmitil inhalation powder-TPIP (prostacyclin analog)	HP-ILD N = 31	Number of participants with TEAEs up to 25 months	2/3	Active, not recruiting	March, 2026
NCT05505409	Pirfenidone (antifibrotic)	CTD-ILD N = 120	Change in % FVC from 6 months to baseline	4	Recruiting	December, 2025
NCT04928586	Pirfenidon combined with IS	CTD-ILD N = 200	Change in FVC and DLCO at 12 months	4	Active, nor recruiting	June, 2025
FIBROPOC (NCT06714123)	Senicapoc (gardos channel blocker)	PF-ILD/IPF N = 140	The rate of decline in ppFVC over 26 weeks	2	Not yet recruiting	December, 2028
NCT05892614	Efzofitimod (tRNA synthetase inhibitor)	SSc-ILD N = 25	Absolute change in FVC at 24 weeks and change in HRCT fibrosis score	2	Recruiting	December, 2024
INTENSE (NCT05503030)	Nintedanib (TK inhibitor)	CTD-ILD N = 87	Change in dyspnea and cough at 24 months	Observational	Observational	December, 2026
NINTOC-TU (NCT06297096)	Nintedanib and Tocilizumab (IL-6 targeting) vs conventional IS	SSc-ILD N = 86	Decrease in FVC after 56 weeks	3	Not yet recruiting	March, 2028
NCT06189495	Genakumab GenSci 048 (BLys/APRIL dual antagonist)	RA-ILD SSc-ILD N = 30	Change in FVC and DLCO up to 2 years	2	Recruiting	October, 2026
ATHENA-SSc-ILD (NCT05270668)	Tulisokibart MK-7240/PRA023 (TNF-TL1A inhibitor)	SSc-ILD N = 152	Number of participants experiencing AEs and change in FVC at week 50	2	Recruiting	June, 2029
NCT05139719	Yfenidone (HEC585) (TGF-α and TGF-β inhibitor—Pirfenidone analog)	PF-ILD N = 110	Change in FVC from baseline to week 24	2	Recruiting	December, 2026

Abbreviations: ILD, interstitial lung disease; IPF, idiopathic lung fibrosis; RTX, rituximab; PF-ILD, progressive fibrosing ILD; (pp)FVC, (percent predicted) forced vital capacity; MMF, mycophenolate mofetil; SSc-ILD, systemic sclerosis-associated ILD; Blys, B lymphocyte stimulator; CTD-ILD, connective tissue disease-associated ILD; JAK, Janus activated kinase; RA-ILD, rheumatoid arthritis-associated ILD; AEs, adverse events; QILD, quantative interstitial lung disease; TK, tyrosine kinase; HA, hyaluronan; HP-ILD, hypersensitivity pneumonitis-associated ILD; SAEs, severe adverse events; LPA, lysophosphatilic acid; SSEs, spontaneous syncopal events; TEAEs, treatment emergent adverse events; DLCO, diffusing-capacity for carbonmonoxide; tRNA, transfer rybonucleic acid; HRCT, high-resolution computed tomography; APRIL, proliferation-inducing ligand; TNF-TL1A, tumor necrosis factor-like cytokine A; TGF, transforming growth factor.

## Data Availability

Not applicable.

## Abbreviations

The following abbreviations are used in this manuscript:

ANCA, anti-neutrophil cytoplasmic antibody; AAV, ANCA-associated vasculitis; GPA, granulomatosis with polyangiitis; MPA, microscopic polyangiitis; EPGA, eosinophilic granulomatosis with polyangiitis; PR3-ANCA, proteinase 3-ANCA; MPO-ANCA, myeloperoxidase-ANCA; DAH, diffuse alveolar hemorrhage; ILD, interstitial lung disease; IPF, idiopathic pulmonary fibrosis; ANCA-ILD, ANCA-positive ILD, AAV-ILD, ILD secondary to AAV; NET, neutrophil extracellular trap; TLS, tertiary lymphoid structure; ENT, ear, nose, throat; RF, rheumatoid factor; PFT, pulmonary function test; TL,co, transfer factor of the lungs for carbon monoxide; UIP, usual interstitial pneumonia; NSIP, non-specific interstitial pneumonia; OP, organizing pneumonia; DIP, desquamative interstitial pneumonia; BALF, bronchoalveolar lavage fluid; TTE, transthoracic echocardiography; 6-MWT, 6 min walk test; GERD, gastroesophageal reflux disease; FVC, forced vital capacity; IS, immunosuppressive; RA, rheumatoid arthritis; PPF, progressive pulmonary fibrosis; IPAF, interstitial pneumonia with autoimmune features; PF-ILD, progressive fibrosing ILD; CS, corticosteroids; CYC, cyclophosphamide; RTX, rituximab; MTX, methotrexate; AZA, azathioprine; MMF, mycophenolate mofetil; CTD, connective tissue disease; SSc, systemic sclerosis; HP, hypersensitivity pneumonitis; JAK, Janus kinase; STAT, signal transducer and activator of transcription; PDE, phosphodiesterase
